# The Impacts of Planting Patterns Combined with Irrigation Management Practices on Watermelon Growth, Photosynthesis, and Yield

**DOI:** 10.3390/plants13101402

**Published:** 2024-05-17

**Authors:** Xiaolin Qiang, Zhaojun Sun, Xingqiang Li, Siqi Li, Zhao Yu, Jun He, Qian Li, Lei Han, Ling He

**Affiliations:** 1 School of Civil and Hydraulic Engineering, Ningxia University, Yinchuan 750021, China; qiangxiaolin123@163.com (X.Q.); 12021140101@stu.nxu.edu.cn (X.L.); siqi7li@163.com (S.L.); nanxia_yu@163.com (Z.Y.); 2 School of Geography and Planning, Ningxia University, Yinchuan 750021, China; hejun3025@163.com (J.H.); li_q@nxu.edu.cn (Q.L.); layhan@163.com (L.H.); 3 China-Arab Joint International Research Laboratory for Featured Resources and Environmental Governance in Arid Region, Yinchuan 750021, China; 4 Key Laboratory of Resource Assessment and Environmental Control in Arid Region of Ningxia, Yinchuan 750021, China; 5 Ningxia Institute of Science and Technology Development Strategy and Information Research, Yinchuan 750021, China; 17795028583@163.com

**Keywords:** agroforestry planting pattern, watermelon sole-cropping pattern, irrigation strategies, yield

## Abstract

(1) Background: Crop yields in China’s arid and semi-arid regions are limited by water shortages. Exploring the interactions and resource utilization among agroforestry species is key to maintaining diversified agricultural production. (2) Objective: An apple–watermelon agroforestry system and watermelon sole-cropping system were compared to quantify how resource availability (light, water) and watermelon performance (leaf photosynthetic rate, growth, and yield) change with irrigation strategies. (3) Methods: A three-year apple and watermelon field experiment was conducted in a young apple orchard in the arid area of central Ningxia to test the effect of light competition and irrigation systems on light environment, leaf photosynthetic rate, plant growth, and yield in watermelon. The experiment encompassed two planting patterns: (i) apple–watermelon agroforestry (AF) and watermelon sole-cropping (SC) and (ii) three irrigation quotas (W1: 105 mm, W2: 210 mm, and W3: 315 mm). (4) Results: The results show that the agroforestry planting pattern extended the growth period of watermelon and increased the leaf area index. Mean daily shade intensity increased by 16.02% from 2020 to 2022. The land equivalent ratio (LER) was >1 in 2021 and 2022. The SWC, leaf photosynthetic rate, LAI, and yield of watermelon in an agroforestry planting pattern were lower than when in a sole-cropping planting pattern. However, under the W1 irrigation strategy, the total soluble solids of the agroforestry planting pattern were 2.27% higher than those of the sole-cropping pattern, and the yield of the agroforestry planting pattern was 2.59% higher than that of the sole-cropping pattern. Under the W3 irrigation strategy, the average watermelon weight in the agroforestry planting pattern was 2.85% higher than that of the sole-cropping pattern. A path analysis showed that the agroforestry planting pattern can increase the yield by increasing soil water content, which is different from the sole-cropping pattern. (5) Conclusions: The results confirm that the apple–watermelon agroforestry planting pattern reduced watermelon yields. However, the LER of the agroforestry system was greater than 1.0. It is reasonable to plant watermelons in young apple forests.

## 1. Introduction

In arid areas, water shortages are threating the sustainability of agriculture. Secondly, the pursuit of higher yields in agricultural production has led to a sharp increase in agricultural investment, which is accompanied by a large amount of wasted water resources and the excessive application of fertilizers. In recent years, many places in China have developed efficient water use as an important measure to solve issues related to “agriculture, rural areas and farmers”. Irrigation plays an important role in regulating plant growth and water utilization, and has a significant regulatory effect on periodic changes in soil moisture. Drip irrigation can inhibit soil water evaporation, achieve precise water and fertilizer regulation [1], and promote the root absorption of limited resources and crop photosynthesis [2]. Studies have shown that drip irrigation can promote the root growth of fruit trees and tomatoes and increase water productivity [3,4].

Agroforestry planting pattern may be a strategy to alleviate low water and land use efficiency. The clearest advantage is that, as a dynamic artificial compound management system, compared with monoculture crops, it plays an important role in reducing soil erosion and improving land use efficiency [5]. Many agroforestry systems at home and abroad have developed into diversified and sustainable land use patterns, but there are different laws of resource allocation and utilization among different regions and species. In tree-based alley cropping systems, where plants co-grow, every plant tries to compete for available resources for its own needs [6]. Resource competition/complementarity between trees and crops is bound to occur. In the aboveground, when the tree overlaps with the crop growth period, the tree height and crown width will affect the light interception of the crop, and the inter-row broadband will affect light transmission in the agroforestry system [7]. Among different production limitations, light availability may be the most significant limitation to the performance of the crops [8], particularly where an upper storey perennial forms a continuous overstorey canopy. In the study of the effects of competition and complementarity of light on plant functional and structural plasticity, it was found that there was little difference in light energy utilization efficiency between legume crops and non-legume crops [9]. Canopy air temperature has a negative correlation with leaf physiological characteristics, plant growth and crop yield [2]. The agroforestry planting pattern will lower the canopy air temperature [10], which can improve the leaf net photosynthesis rate, chlorophyll concentration [11] and leaf water use efficiency of the plants under the tree canopy, thus benefiting crop photosynthesis and dry matter accumulation. In the subterranean realm of the agroforestry planting pattern, roots intertwine and engage in subterranean interactions. Varied water irrigation demands and the distinctive characteristics of tree and crop root growth and distribution may induce water migration, thereby enhancing the water-sharing mechanism and redistributing water within the soil profile. This approach represents a promising strategy for adapting to drier conditions and sustaining yield productivity. The realm of agriculture has witnessed a plethora of studies on intercropping, with widespread acknowledgment of the significance of species interaction in soil nutrient dynamics [12]. Certain studies have indicated a significant increase in soil nitrate concentration after three years of agroforestry treatment [13], which did not decrease olive yield.

Selecting annual crops for cultivation in the spaces between fruit trees has the potential to enhance light interception in the lower canopy, mitigating issues like excessive competition, nutrient deficiency, and water scarcity between mature fruit trees and crops. This approach addresses concerns related to the internal environment and soil conditions within the agroforestry system. The interplay of diverse root configurations and intercropping density may facilitate lateral water flow from the root zone of one species to another [14], thereby augmenting soil moisture for crops adjacent to trees. Additionally, trees contribute to the microclimate by casting shadows, influencing factors like relative humidity, which, in turn, can reduce evapotranspiration, resulting in a positive impact on soil moisture. The intercropping model’s deeper roots and enhanced water storage capacity in trees may alleviate the impacts of drought. Nevertheless, numerous studies have indicated that the presence of trees can constrain inter-row crop productivity and potentially worsen yield losses during drought conditions. Despite decades of research, comprehensively evaluating the competitive dynamics between intercropped crops and dominant tree species remains a formidable challenge for agricultural ecologists [15]. The land equivalent ratio (LER) is generally considered to be an indicator for studying whether there is a competitive relationship between species in agroforestry planting patterns. If the value is greater than 1, the pattern is considered to have improved land use efficiency [16].

Watermelon (*Citrullus lanatus*) stands out as a drought-tolerant fruit, boasting commendable economic returns. The arid expanse of central Ningxia is characterized by abundant light and heat resources, featuring a meager average annual precipitation of merely 150 mm and staggering evaporation rates reaching up to 2200 mm. This region proudly holds its status as a primary watermelon production hub in China. However, the juxtaposition of watermelon cultivation with fruit trees in agroforestry poses a challenge due to the high light and water requirements essential for optimal watermelon growth. There are few studies on the efficient resource utilization of combining subsurface drip irrigation and agroforestry planting patterns. Furthermore, it is difficult to predict whether the water and crown radiation capture of fruit tree growth with increasing years will affect the yield and quality of watermelon in agroforestry planting patterns. Furthermore, there are few studies that investigate the synergies arising from efficient resource utilization through the integration of irrigation levels and agroforestry planting patterns, with challenges in predicting long-term outcomes. Studies have showed that planting crops between apple tree rows proves to be an approach to enhance the economic benefits of young orchards and land productivity in arid regions, especially before apple trees reach the fruiting stage [17]. However, it remains uncertain whether the water uptake by fruit trees and the canopy’s radiation interception impact the yield and quality of crops in agroforestry settings as the years progress. In comparison to monoculture, the optimistic expectation is that young apple trees and intercropped watermelons can consistently optimize the sharing of limited light and water resources through temporal and/or spatial resource allocation. The interplay between watermelon and young apple trees has the potential to enhance the efficient utilization of soil water, nutrients, and solar radiation, contributing to an overall improvement in yield. In essence, delving into the mechanics of how the scarcity of light and water influences the interaction between these two species in an agroforestry system is crucial for comprehending the dynamics of agroforestry.

To realize efficient land and water utilization in arid regions, this study delves into the impacts of various irrigation strategies and planting configurations on the overall soil environment for watermelon cultivation and the yields of both watermelon and apple trees. We aim to explore the repercussions of irrigation and intercropping scenarios on the light and water stresses experienced by watermelon plants, impacting leaf photosynthetic characteristics, overall plant growth, and watermelon yield over three consecutive observation years. Through quantitative analysis, we examine the responses of key factors such as net photosynthetic rate, chlorophyll concentration, plant growth, and watermelon quality to variations in a soil–agroforestry intercropping system, shedding light on the intricate interplay between water and light intensity in the context of this agricultural ecosystem.

## 2. Materials and Methods

### 2.1. Site Description

This field study was conducted at the Dry Farming Water-saving Agricultural Science and Technology Park in Wangtuan Town (36°50′ N, 105°60′ E), Tongxin District, Wuzhong City in the Central arid zone of Ningxia. The annual temperature is 8.7 °C. The annual precipitation is 270 mm, with 60–70% occurring from July to September. The average annual pan evaporation is 2325 mm.

**Figure 1:**
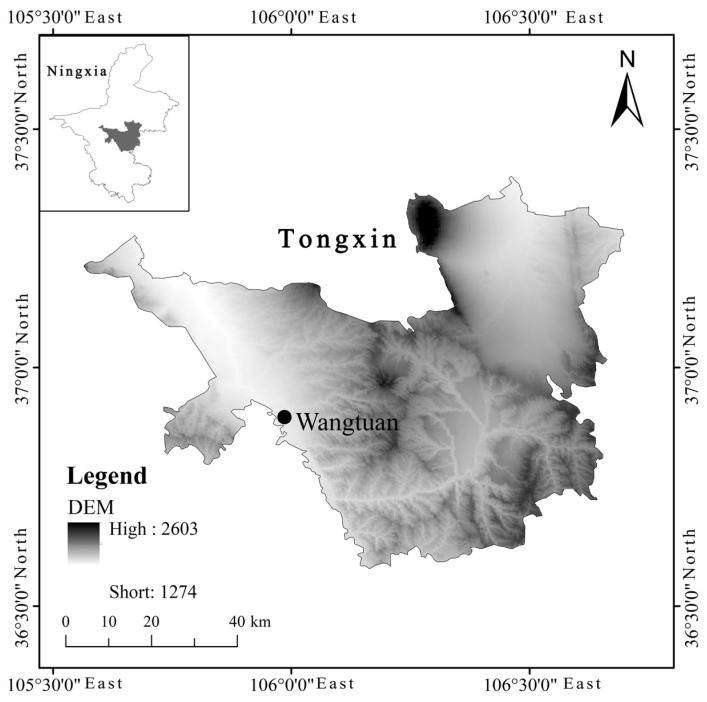
Location of this study.

Figure 1 shows an overview of the study area.

### 2.2. Experimental Design

The drip type had a dripper spacing set at 0.2 m with a flow rate of 2.0 L h^−1^, and it operated under a working pressure of 0.1 MPa. Each plot was equipped with a water meter (LXS-25, Ningbo, China), pressure meter, and control valve to ensure accurate discharge and pressure stability.

The experiments were conducted in a randomized block factorial design with three replicates, and the treatments were repeated at the same site in 2020, 2021 and 2022. The experimental treatments comprised (1) two planting patterns (apple–watermelon agroforestry planting pattern (AF) and watermelon sole-cropping pattern (SC)) and (2) three watermelon irrigation strategies (W1: 105 mm, W2: 210 mm, and W3: 315 mm). The irrigation quota is shown in Table 1.

**Figure 2:**
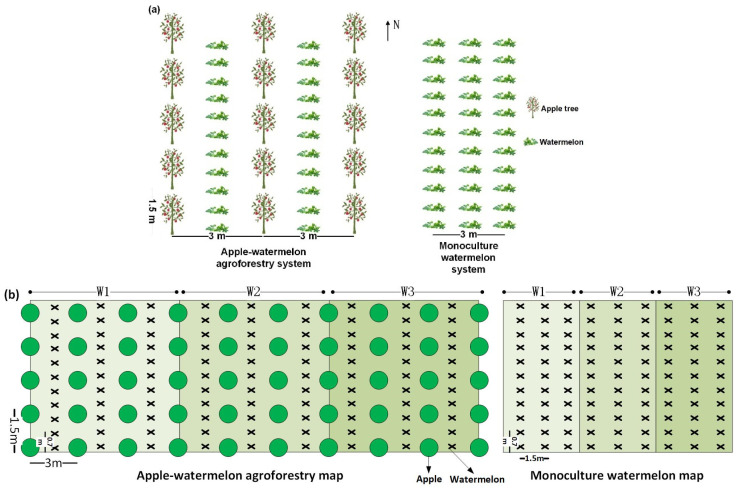
The layout design of row spacing of the apple–watermelon agroforestry system and watermelon sole-cropping system: (**a**) plot and block design map; (**b**) the planting area of watermelon in agroforestry system is half of that in the watermelon sole-cropping system.

There was a total of 18 plots, with each plot measuring 6.0 m × 3.0 m for the agroforestry system and 6.0 m × 1.5 m for the watermelon sole-cropping system. Each plot has 3 replicates. The agroforestry plots comprise an area occupied by 10 apple trees configured as 2 rows × 10 trees within rows. Tree spacing was 6.0 m between rows and 3.0 m between trees within rows. Watermelons in the agroforestry system were planted in a single row parallel to two rows of trees, with a distance of 70 cm between plants. For the watermelon sole-cropping pattern, there were also 9 plots. The plant spacing in this system was 0.7 m × 1.5 m. The layout design of row spacing is shown in Figure 2.

**Figure 3:**
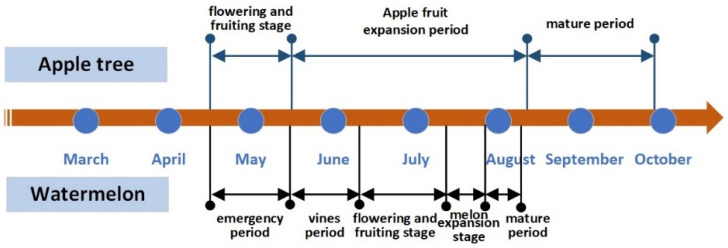
The co-growth timeline of apple tree and watermelon in the agroforestry system.

In the agroforestry planting pattern, Michaela is the variety of apple trees. The 3-year-old apple trees were planted in 2019, with average heights of 1.9 m, 2.2 m, and 2.5 m in 2020, 2021, and 2022, respectively. The watermelon variety was Jincheng No. 5 and seedlings were planted on 20 April 2020, 17 April 2021 and 18 April 2022, respectively, with harvest starting on 28 July 2020, 18 July 2021 and 20 July 2022. The co-growth timeline of apple tree and watermelon in the agroforestry system is shown in Figure 3.

### 2.3. Data Collection

#### 2.3.1. Soil Water Content Measurement

A soil core sample (5 cm internal diameter) was used to measure the soil water content before and after irrigation and rainfall at observation points at soil depths of 0–10 cm, 10–20 cm, 20–40 cm, 40–60 cm, 60–80 cm, and 80–100 cm.

#### 2.3.2. Photosynthetic Parameters

The net photosynthesis rates (Pn, µmol·m^−2^s^−1^) of the watermelon leaves were measured with a portable photosynthetic system (Li-6400, LI-COR Inc., Lincoln, NE, USA) in clear and cloudless weather during the flowering and fruit setting stage and melon expansion stage. We randomly selected the 5th to 7th functional leaves from top to bottom from 5 well-growing watermelon plants. The photosynthetically active radiation (PAR, µmol·m^−2^s^−1^) at this location was recorded at the same time by Li-6400 portable photosynthetic system. Measurements were made once every 2 h between 8:00 and 18:00. To characterize the radiation environment at different locations in the canopy and at different times of the day.

The average shade intensity under agroforestry planting pattern was calculated using the following formula.

The mean daily shade intensity was calculated by:

(1) $$\mathrm{Mean}\;\mathrm{daily}\;\mathrm{shade}\;\mathrm{intensity}\;(\%)=({\mathrm{PAR}}_{\mathrm{MONO}}-{\mathrm{PAR}}_{\mathrm{INT}})/{\mathrm{PAR}}_{\mathrm{MONO}}\times 100\%$$

where PAR_mono_ is the mean daily PAR of the watermelon sole-cropping system; PAR_int_ is the mean daily PAR of the agroforestry system.

#### 2.3.3. Watermelon Growth Characteristics Indicators

Watermelon vine length was measured using a measuring tape; the stalk thickness near the root of the main vine was measured using a digital vernier caliper. To ensure that the same position was selected each time, the selected plants to be measured needed to be marked, and three plants were randomly selected for each treatment.

#### 2.3.4. Yield and the Soluble Solids (TSS) of Watermelon

Fruits were harvested twice: on 21 August 2020 and 4 September 2020, on 26 August 2021 and 5 September 2021, and on 30 August 2022 and 9 September 2022. The total soluble solids (TSS) of mature watermelons were measured three times using a handheld refractometer.

#### 2.3.5. Leaf Area Index

The leaf area was calculated by measuring the length and width of the blade with a steel tape measure, and then calculated as follows:

(2) $$S_{\mathrm{leaf}}=L_{\mathrm{leaf}}\times H_{\mathrm{leaf}}\times 0.75$$

(3) LAI=SleafSground

#### 2.3.6. LER

Conceptually, the land equivalent ratio (LER) represents the relative land area required to achieve the same yield or biomass per unit area as a single crop in an intercrop. It is expressed as follows [18]:

(4) $$\mathrm{LER}=\frac{Y_{\mathrm{AF}-W}}{Y_{\mathrm{SC}-W}}+\frac{Y_{\mathrm{AF}-A}}{Y_{\mathrm{SC}-A}}$$

where Y_AF-W_ and Y_AF-A_ are the yields of the watermelon and apple tree in the agroforestry system, respectively; Y_SC-W_ and Y_SC-A_ are the productivity of the watermelon and apple tree in the sole-cropping system. When LER > 1, there is a land use advantage of the agroforestry system; if LER = 1, there is no productive advantage, and if LER < 1, there is no advantage to using the agroforestry system. The calculation of LER is mainly based on the yield per unit area, under the condition of ensuring the same planting density.

### 2.4. Statistical Analysis

Differences between the treatments were examined using analysis of variance (ANOVA) and least significant differences (LSDs). We analyzed differences in soil water content (SWC), leaf net photosynthetic rate (pn), chlorophyll content (SPAD), leaf area index (LAI), yield, and other growth parameters among six treatments. In the general linear model, planting pattern (M), irrigation strategy (W) and their interaction (M × W) were considered as fixed effects, and Tukey’s honestly significant difference (HSD) was used for the post hoc test. Significant effects were determined as *p* < 0.05. A correlation analysis quantified the correlation coefficient between factors. A path analysis further estimated the direct and indirect effects of each factor on watermelon yield in the AF system and SC system, respectively. The statistical analyses were conducted using SPSS 25.0 software (IBM Corp., Armonk, NY, USA). All graphical representations were generated using OriginPro 2021 software (OriginLab Software Inc., Northampton, MA, USA).

## 3. Results

### 3.1. Light Interception and Photosynthetic Rate

Observations were made on the temporal changes in photosynthetically active radiation (PAR) and shading intensity across the agroforestry planting system. The diurnal PAR variation in both sole-cropping and agroforestry systems consistently peaked at around noon. Over the years, the increasing canopy cover density of apple trees led to a continuous reduction in the PAR reaching the watermelon canopy. Mean daily shade intensity increased by 16.02% from 2020 to 2022. In 2021 and 2022, under the same irrigation strategies, the net photosynthetic rate (Pn) of the sole-cropping planting pattern was significantly higher than that of agroforestry planting pattern (*p* < 0.05). The daily Pn variation typically displayed a double-peak curve, reaching its maximum around 10:00 in 2020 and 2021. As time went on, the angle of sunlight hitting the plants increased, resulting in a decrease in both PAR and Pn, especially noticeable under intercropping conditions, causing a midday depression of photosynthesis before 14:00. However, in 2022, the diurnal Pn variation of the agroforestry treatment showed a single peak. The Pn was relatively high with the W2 irrigation strategy, indicating that excessively low or high irrigation is not favorable for optimal Pn.

**Figure 4:**
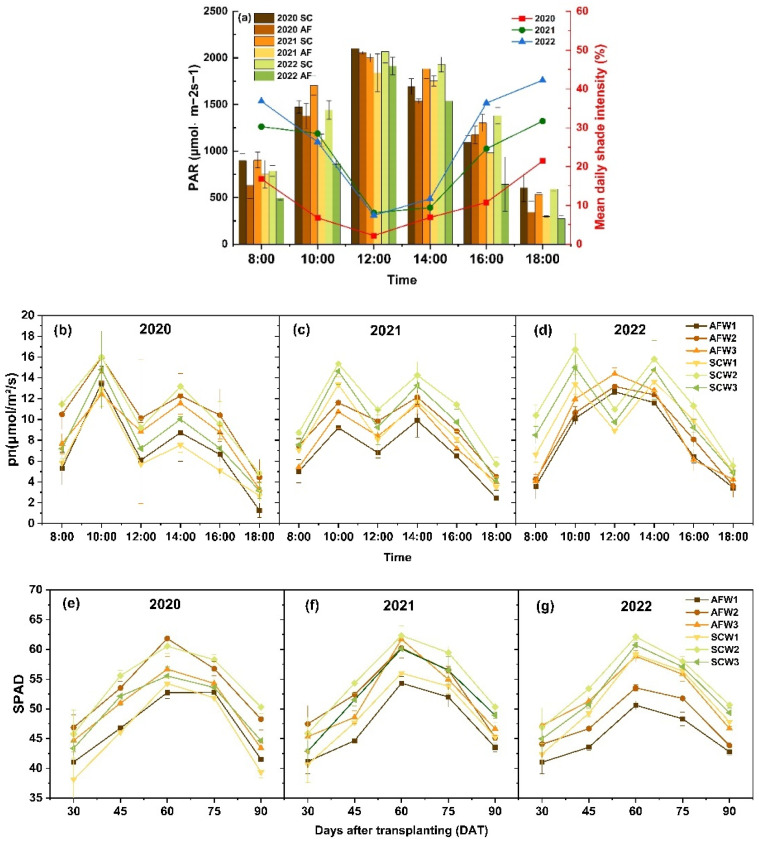
Effects of planting pattern on PAR and mean daily shade intensity of watermelon canopy: (**a**) the diurnal variation in the leaf photosynthetic rate (Pn) of watermelon (**b**–**d**) and chlorophyll content index (SPAD) of watermelon in the fruit expansion stage (**e**–**g**) in 2020, 2021, and 2022.

As shown in Figure 4, there is no significant difference in the SPAD of watermelon leaves under different treatments at the seedling stage. An analysis of variance showed that the relative chlorophyll content of leaves had an insignificant response to different planting patterns in 2020, but a significant response to the amount of irrigation water; in 2022, the planting pattern had a significant impact on chlorophyll content (Table 2). The peak value of chlorophyll content occurs in the late stage of flowering and fruit setting (DOY 60). From the peak of the flowering and fruit setting period to the maturity stage of watermelon in 2022, the chlorophyll content of watermelon in the sole-cropping planting pattern decreased by 10% more than that in the agroforestry planting pattern.

### 3.2. The Soil Water Content in the 0–100 cm Soil Layer

**Figure 5:**
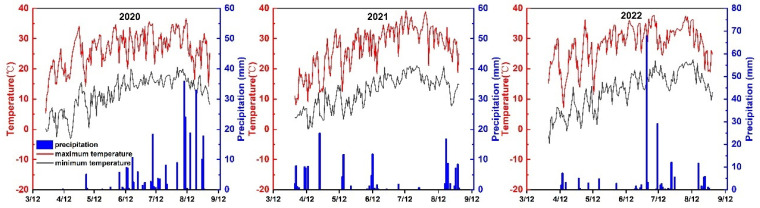
Experimental site meteorology.

During the watermelon growth period, the total precipitation amounted to 156 mm, 192 mm, and 107 mm during the plant growing seasons in 2020, 2021 and 2022. The mean maximum and minimum temperatures recorded were 28.7 °C and 15.1 °C in 2020, 28.3 °C and 14.3 °C in 2021, and 26.9 °C and 13.1 °C in 2022 (Figure 5). Table 3 illustrates the variations in soil water content at a 0–100 cm depth during the flowering and fruit setting stage and melon expansion stage, encompassing the average values for the 0–20 cm, 20–40 cm, 40–60 cm, 60–80 cm, and 80–100 cm layers, which are the average values for 2020, 2021, and 2022. Planting pattern significantly influenced soil water content in each soil layer during the flowering and fruit setting stage (*p* < 0.05). Throughout the three years, the interaction between planting patterns and irrigation strategies had no significant effect on the SWC (*p* > 0.05). The SWC in the 0–20 cm, 20–40 cm, and 80–100 cm soil layers has significant differences between the years at the flowering and fruiting stages, and the SWC in the 0–20 cm and 20–40 cm soil layers also has significant differences at the melon expansion stage. Under the same irrigation strategy, the soil water content in the 0–20 cm soil layer under an agroforestry system was higher than that of the sole-cropping pattern in 2020. At the flowering and fruit setting stage, the SWC in the 20–40 cm soil layer with AFW1 treatment was higher than that with the SCW1 treatment.

### 3.3. Watermelon Plant Growth

**Figure 6:**
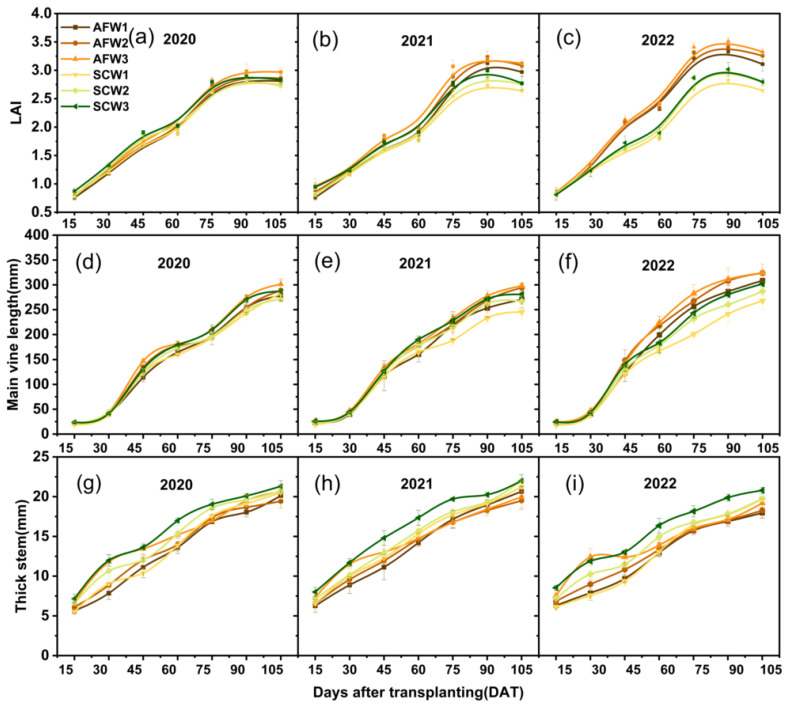
Effect of planting patterns and irrigation strategies on leaf area index (LAI) (**a**–**c**), main vine length (**d**–**f**) and thick stem (**g**–**i**) in 2020, 2021, and 2022.

The leaf area index (LAI) in the agroforestry system for 2021 (*p* < 0.05) and 2022 (*p* < 0.01) exhibited a significant increase compared to 2020. Under the same irrigation strategy, agroforestry treatments demonstrated higher vine length and LAI compared to sole-cropping treatments in 2021 (*p* < 0.05) and 2022 (*p* < 0.01). In 2020 and 2021, under the W2 irrigation strategy, the watermelon vine length is greatest in the agroforestry system; however, in 2022, it occurs under the W3 irrigation strategy. In 2021, following the fruit expansion period of watermelon, the LAI of watermelon treated with the AFW3 treatment exhibited the fastest increase, significantly differing from all monoculture treatments (Figure 6).

There was a significant difference in vine length between agroforestry and sole-cropping watermelon in both 2021 (*p* < 0.05) and 2022 (*p* < 0.01) (Table 4). Vine length exhibited an increasing trend with rising irrigation strategies. In contrast to vine length, the thick stem diameter of the AFW3 treatment experienced accelerated growth before the flowering and fruiting stage but subsequently showed slower growth compared to the W1 and the W2 irrigation strategies. Water deficiency during the flowering and fruiting stages significantly impeded the growth of the main watermelon vine, with the degree of impact escalating with the severity of water deficit. 

### 3.4. Watermelon Yield and Fruit Total Soluble Solids

The results presented in Table 5 indicate that, in the sole-cropping planting pattern, the W2 irrigation strategy achieved the highest yield over the three experimental years. In comparison to sole cropping, the watermelon yield in the agroforestry planting pattern showed a declining trend annually. However, under the W1 irrigation strategies, the yield in the agroforestry system did not significantly differ from that in sole-cropping and in fact surpassed the sole-cropping planting pattern in 2020. In sole-cropping, the average weight of watermelon fruits experienced a sharp decline in 2022. Notably, the AFW3 treatment showed significantly higher individual fruit average weight than that of the SCW3 treatment in 2022 (Table 5). Furthermore, the individual fruit weight exhibited a noticeable increase with improved irrigation levels. Conversely, total soluble solids (TSS) decreased with the rise in irrigation levels.

Table 6 shows the land equivalent ratio (LER) of the last two experimental years. Due to the transplanting and maintenance of apple trees in 2020, the apple trees did not bear fruit and had no yield. The LER value was higher than 1 under each irrigation strategy in the next two years and increased with the increase in irrigation strategy; land productivity also improved.

### 3.5. Correlation and Path Analysis

**Figure 7:**
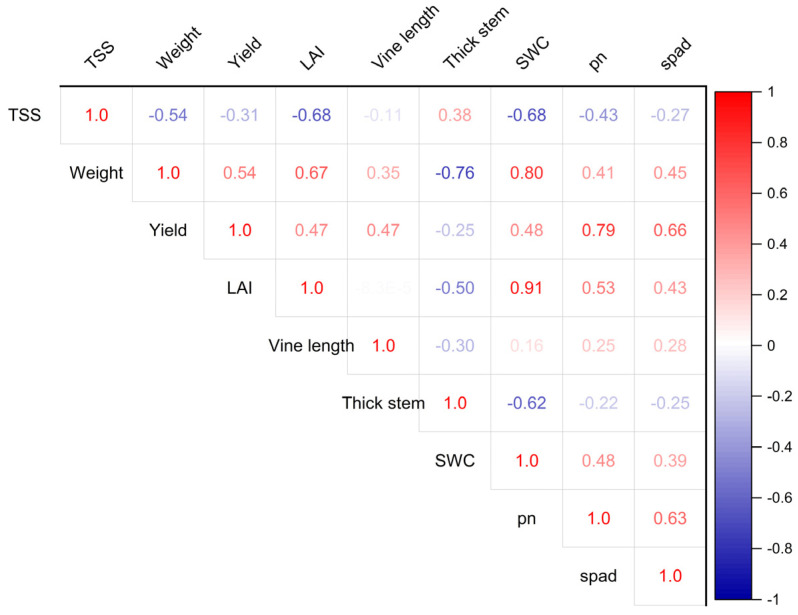
Pearson correlation analysis between watermelon yield, soil water content and other agronomic traits. A table of correlation coefficients is plotted at the top right of the correlation matrix, and a significance analysis is plotted on the opposite side. Shades and sizes of colored circles indicate the degree of correlation.

**Figure 8:**
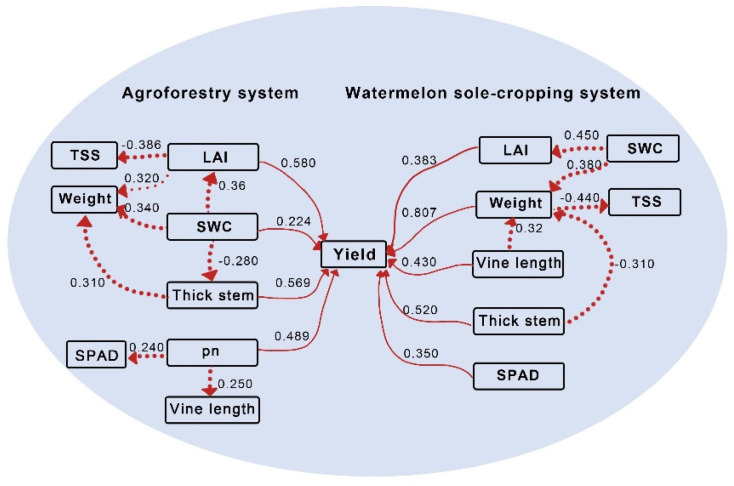
Path coefficients between yield, weight, vine length, thick stem, SWC, and TSS under agroforestry planting pattern and sole-cropping pattern. A diagram demonstrates the causal connection between the yield and five other factors. The arrows indicate the direction of the cause-and-effect relationship between the indices, with solid lines showing the direct path coefficients and dotted lines showing the indirect path coefficients.

The results of the Pearson correlation analysis revealed a positive correlation between soil water content (SWC) and fruit weight (r = 0.80), leaf area index (r = 0.91), and Pn (r = 0.48), SPAD (r = 0.39), while total soluble solids (TSS) and soil water content (r = −0.68), weight (r = −0.54), LAI (r = −0.68), Pn (r = −0.43), and SPAD (r = −0.27) exhibited significant negative correlations (Figure 7). To investigate the inhibitory impact of planting patterns on watermelon yield, a path analysis was conducted to evaluate the direct and indirect effects between yield and other biological characteristics, for example TSS and LAI. Within the watermelon sole-cropping system, LAI, SWC, thick stem, and pn were directly related to watermelon yield (Figure 8). Within the agroforestry planting pattern, LAI, weight, vine length, thick stem, and SPAD were directly related to yield. Notably, SWC also influenced average fruit weight, LAI, and stem diameter under agroforestry planting pattern, thereby contributing to increased yield. This underscores the significant role of SWC regulation in agroforestry.

## 4. Discussion

### 4.1. The Effect of the Agroforestry Planting Pattern on the Photosynthetic Characteristics of Watermelon

During the watermelon expansion period, robust photosynthesis and accelerated organic matter synthesis were evident. The shade provided by apple trees reduces both direct and indirect radiation reaching the soil from the air. Physiological factors such as leaf photosynthesis and chlorophyll concentration are highly sensitive to water and light intensity stress [19]. Chlorophyll concentration, a crucial pigment for light absorption, transfer, and conversion in photosynthesis, reflects the strength of crop photosynthesis to some extent. A higher SPAD value is beneficial for biomass production [20,21]. In 2022, the net photosynthetic rate of leaves was significantly lower in the intercropping planting pattern compared to the monoculture. In the third year, the leaf area index (LAI) of apple trees reached its maximum, significantly impacting watermelon leaf photosynthesis. However, other studies have shown that the presence of trees increases diffuse radiation [9]. This study found that ample irrigation has a mitigating effect on watermelon photosynthesis and SPAD, especially in the heavily shaded conditions of 2022. The net photosynthetic rate (pn) of AFW3 treatment in 2022 showed no significant difference from SCW3 treatment (Figure 5), indicating that sufficient irrigation can alleviate light and temperature stress, reducing the synthesis of abscisic acid (ABA) in lower canopy leaves, thereby limiting the decline of stomatal conductance and transpiration rate [22], thus regulating plant photosynthetic physiological activities. In contrast to another study on wheat intercropping that light utilization is not influenced by irrigation levels [23].

In 2022, the diurnal variation of photosynthetic rate in the AFW3 treatment exhibited a single-peak curve, possibly due to excessive shading causing stomatal closure and restricted activity, hence no midday depression of the photosynthesis of watermelon. The SPAD in the agroforestry system show a smaller decrease throughout the growth period compared to the sole-cropping system. Under the agroforestry system, watermelon leaves, in order to maintain higher chlorophyll content, extended their photosynthesis, providing sufficient nutrients for the watermelon’s fruit expansion period. Additionally, the shade from apple trees reduced the canopy temperature of watermelon, lowering crop respiration, and thereby maintaining photosynthesis and biomass accumulation in crops [24,25]. This is consistent with studies suggesting a negative correlation between chlorophyll content and canopy temperature [26].

### 4.2. The Effect of Agroforestry Planting Patterns and Irrigation Strategies on Vertical Distribution of Soil Water Content

In this study, the soil water content in 2022 was lower than in the previous two years, probably because the precipitation in 2022 was less than that in the previous two years. Soil moisture content serves as an indicator of environmental stress and the impact of species interactions on watermelon growth. In semi-arid regions, studies have shown that trees and crops can compete for water, especially under conditions of sufficient moisture, as both trees and crops tend to absorb water from the shallow soil layers [27]. During the watermelon flowering and fruit setting stage, the SWC of the AFW1 treatment and SCW1 treatment has no significant difference, possibly because the apple trees did not have deep roots and consumed less water. When apple trees began to sprout, the watermelon roots continuously extended further. The water uptake of both species depends on the ability of their roots to produce potential gradients in soil water [28].

Furthermore, this study demonstrates that tree rows are advantageous for soil moisture retention under certain irrigation conditions. Although trees have been proven to reduce incoming net photosynthesis effective radiation, relatively high humidity, lower wind speed, and direct sunlight can help reduce soil evaporation within the agroforestry planting system. During the watermelon flowering and fruit setting stage, the 20–40 cm soil layer SWC of the AFW1 treatment was higher than that of the SCW1 treatment, possibly because (1) apple trees alter their water uptake targets in the surface soil during periods of soil drought, alleviating crop water pressure [29], and (2) in the third year of apple tree transplantation, the large leaf area of apple trees created a microclimate under the canopy, reducing air temperature and soil evaporation, which may have increased the available water for watermelon. Moreover, in the 20–40 cm soil layer, the difference between planting patterns increased with the increase in irrigation strategies (W). Plant interspecific interactions in drought years may allow fruit trees to extract water from deeper soil layers, while in wet conditions, creating more intense competition for shallow soil moisture with the increased amount of irrigation [30]. However, the positive aspect is that lower soil water content can stimulate the extension and growth of fine watermelon roots [31].

### 4.3. The Effect of Agroforestry Planting Patterns and Irrigation Strategies on Watermelon Growth

The climatic conditions during the flowering and fruit setting stage are crucial for watermelon growth, and they are also one of the key considerations for researchers assessing the suitability of the coexistence of different crops and tree species [32]. In this study, during the flowering and fruit setting stage of watermelon, apple trees were in the budding stage and had not yet cast a canopy shade on the inter-row watermelon, thereby not reducing temperature and photosynthetically active radiation. Consequently, there were no significant differences in stem diameter, vine length, and LAI among different planting patterns during this period. One of the advantages of planting in the tree rows is that watermelon has a lower light saturation point, exhibiting lower sensitivity to shading. In the third year of agroforestry observed in this study, shading negatively affected the growth parameters of watermelon, extending the melon’s expansion and maturation period compared to sole-cropping system. The increase in LAI in 2022 is a response of watermelon to shading, aiming to enhance radiation interception efficiency and increase its photosynthetically active surface [33]. In 2021 and 2022, under W1 treatment, watermelon growth parameters of the agroforestry planting pattern exceeded those of the sole-cropping planting pattern, as alternating soil moisture under drought conditions and reduced canopy temperature promoted the growth (leaf area index and vine length) of watermelon. This outcome suggests that shading can improve the thermal environment of water and promote plant growth when soil water is limited. With the increasing number of years, it is advisable to plant watermelon slightly earlier to minimize the coexistence period of the two species. Furthermore, adjusting the compound design through various management measures, such as root pruning, canopy pruning [34], and appropriately reducing the planting density of intercropped watermelon, can alleviate the negative impact of mature orchards becoming dominant species.

### 4.4. The Effect of Agroforestry Planting Patterns and Irrigation Strategies on Watermelon Yield

In 2021 and 2022, the yield in the agroforestry planting pattern was significantly lower compared to the sole-cropping pattern. This difference can be mainly due to the shading, which led to the robust vegetative growth of watermelon plants, consequently hampering their reproductive growth. Surprisingly, with the W1 irrigation strategy, the watermelon yield in agroforestry system surpassed that of sole-cropping system. This is attributed to the apple tree rows in the agroforestry system reducing crop transpiration and soil water evaporation, and thus mitigating drought stress [35]. Additionally, the root system of fruit trees plays a role in stabilizing shallow soil water, compensating for the negative impact of lower irrigation strategies.

Theoretically, crop growth can reach it maximum when all resources are equally limited [36]. The concentration of rainfall during the watermelon expansion period in 2022, coupled with insufficient sunlight and reduced temperature variation, negatively affected watermelon growth and nutrient accumulation. To deal with the shading effects of apple trees, watermelon prolonged leaf photosynthesis time and growth period, attempting to enhance light capture, yet struggling to counteract the negative influence on yield. Watermelon, having longer lateral roots compared to other crops, benefits from the warming and moisture-retaining effect of plastic mulch [37], facilitating root extension. Watermelon roots respond to water scarcity by compensatory growth, absorbing moisture and nutrients from a greater distance, explaining the comparable yield under W1 agroforestry planting pattern. Differences between years may be due to the effects of rainfall and high temperatures. The average watermelon weight has dropped significantly in 2021, which may be due to the influence of high-temperature weather during the watermelon expansion period, compared with 2020 and 2022. However, it can be assumed that planting watermelons under apple trees can slightly resist high temperatures in summer, as well as the diseases and insect pests that are easily brought about by high temperatures and drought.

Since transplanted apple trees were maintained before 2021 to prevent fruit bearing from causing strong competition with apple tree growth nutrients, picked the apple’s flowers and had no yield in 2020, thus LER cannot be calculated. From the LER values from the last two years, it can be found that the apple–watermelon agroforestry system can achieve better land use in semi-arid areas because the understory area is developed and watermelon grows well. According to the apple orchards, watermelon as a cover plant can improve land utilization and economic benefits, and studies have proven that plant covers can reduce soil evaporation in semi-arid areas [38].

Environmental stress, such as water and light intensity, reduces leaf photosynthesis, impacting plant growth and crop productivity, ultimately reducing crop yield [39]. Photosynthesis correlates positively with chlorophyll content in the agroforestry planting pattern. To enhance its photosynthetic activity, watermelon increases the chlorophyll content of its leaves between the apple tree rows to adapt to changing environmental conditions. The available yield not only depends on the total biomass produced by photosynthesis but also on the nutrient distribution in plant organs resulting from photosynthesis. Previous studies indicate that under light stress, tomatoes adjust stem and lead growth rather than fruit growth, which may favor leafy vegetables but decrease yield in fruit-bearing crops. Soil moisture had the most significant direct effect on the photosynthetic rate [40]. In our study, there was a significant interaction between planting patterns and irrigation levels (*p* < 0.05). A path analysis revealed that thick stems, vine length, LAI, and soil water content (SWC) were the primary influencing factors on watermelon yield under the agroforestry planting pattern. Moreover, the increase in single fruit weight was attributed to enhanced LAI, SWC, and pn, confirming that this planting pattern can improve watermelon yield by increasing SWC.

## 5. Conclusions

The agroforestry system and irrigation levels exerted varying effects on watermelon photosynthesis, growth, soil water content, and yield over three consecutive research years. We quantified the spatiotemporal resource capture of watermelon, demonstrating complementary interactions between species in both temporal (different phenology) and spatial (soil moisture) aspects.

The agroforestry planting pattern increased 0–20 cm soil water content by reducing soil evaporation through water movement and shading effects. This pattern prolonged the watermelon growth period, enhancing photosynthesis time, facilitating the efficient utilization of both strong and weak light, resulting in increased single fruit weight and total soluble solids (TSS) but reduced yield. However, under the agroforestry planting pattern, the W1 irrigation strategy promoted water complementarity between apple trees and watermelon, enhancing drought resistance in the agroforestry system. Additionally, under the high-temperature and drought environments of 2021, shading may have been beneficial to the growth of watermelons. In 2022, the W3 irrigation strategy significantly increased average fruit weight. Thus, the agroforestry planting pattern may counterbalance the negative impact of insufficient irrigation on watermelon production.

## Figures and Tables

**Table 1:** The irrigation quota of watermelon.

Watermelon Growth Period	Irrigation Date	Irrigation Quota of Watermelon/mm		
		W1	W2	W3
Seedling stage	17 April	15	30	45
Vines period	13 May	15	30	45
	24 May	15	30	45
Flowering and fruiting stage	8 June	15	30	45
	17 June	15	30	45
	27 June	15	30	45
Melon expansion stage	7 July	15	30	45

**Table 2:** ANOVA on the GLM model for photosynthetic rate (Pn) and chlorophyll content (SPAD) as a function of the year (Y), irrigation strategies (W), planting pattern (M), and irrigation strategies × planting pattern (W × M).

Test of Significance (*F* Value)						
Treatment	Pn			SPAD		
	2020	2021	2022	2020	2021	2022
Year (Y)	48.341 **	7.934 *	0.741	0.898	6.291 *	8.348 **
Irrigation (W)	8.394 **	24.385 **	9.660 *	15.858 **	4.000 *	43.548 **
Planting pattern (M)	33.213 **	19.00 **	16.486 *	23.539 *	33.985 **	18.485 **
W × M	0.557	1.373	0.610	0.501	1.208	1.060

* indicates significance at *p* level of 0.05, and ** indicates significance at *p* level of 0.01.

**Table 3:** Soil water content (SWC) in the 0–100 cm soil layer affected by year (Y), planting patterns (M) and irrigation strategies (W) and their interaction (W × M) at the flowering and fruiting stage and melon expansion stage in 2020, 2021, 2022. Different letters indicated significant differences among different treatments (*p* < 0.05) using an LSD test.

	The Soil Water Content (%)									
	Flowering and Fruiting Stage					Melon Expansion Stage				
Treatment	0–20 cm	20–40 cm	40–60 cm	60–80 cm	80–100 cm	0–20 cm	20–40 cm	40–60 cm	60–80 cm	80–100 cm
AFW1	12.91 ± 0.8 d	12.03 ± 0.58 c	12.85 ± 0.78 c	13.57 ± 1.01 cd	14.05 ± 0.45 cd	12.26 ± 0.77 c	11.08 ± 0.79 c	12.15 ± 0.85 c	13.13 ± 0.95 c	13.06 ± 0.8 c
AFW2	13.76 ± 0.56 c	12.49 ± 0.46 c	13.6 ± 0.66 b	14.26 ± 0.87 c	14.8 ± 1.34 c	13.21 ± 1.36 b	11.72 ± 0.59 c	12.8 ± 1.1 c	13.57 ± 0.71 bc	14.07 ± 1.04 bc
AFW3	14.56 ± 0.64 b	13.47 ± 0.98 b	14.39 ± 1.06 ab	15.16 ± 0.71 b	15.73 ± 0.76 b	13.71 ± 0.4 ab	12.57 ± 1.11 b	14.03 ± 0.85 a	14.06 ± 0.68 b	14.59 ± 0.93 b
SCW1	13.02 ± 0.51 d	11.94 ± 0.63 c	13.69 ± 0.86 b	14.09 ± 0.6 c	14.8 ± 0.8 c	12.78 ± 0.37 bc	11.99 ± 0.81 bc	13.01 ± 0.88 b	13.35 ± 1.43 c	14.03 ± 1.05 bc
SCW2	14.17 ± 0.35 b	13.3 ± 0.8 b	14.68 ± 1.09 ab	15.24 ± 0.54 b	15.98 ± 1.26 b	14.14 ± 0.65 a	12.53 ± 0.76 b	13.46 ± 0.54 b	14.06 ± 0.65 b	15.09 ± 0.93 ab
SCW3	15.06 ± 0.98 a	14.21 ± 0.59 a	15.25 ± 0.84 a	16.37 ± 0.54 a	16.7 ± 0.73 a	14.76 ± 0.93 a	13.37 ± 0.78 a	14.52 ± 0.63 a	15.16 ± 0.69 a	15.82 ± 1.16 a
**Test of Significance (*F* Value)**										
Year (Y)	3.08 *	7.934 *	0.741	0.898	6.291 *	8.348 **	5.763 **	1.035	0.604	15.554 *
Irrigation (W)	13.222 **	24.385 **	9.660 *	15.858 **	4.000 *	43.548 **	12.133 **	14.539 **	10.099 *	14.352 *
Planting pattern (M)	33.213 **	19.00 **	16.486 *	23.539 *	33.985 **	18.485 **	23.495 **	19.43 **	3.859	21.433 *
W × M	0.557	1.373	0.610	0.501	1.208	1.060	0.682	1.554	1.028	0.812

* indicates significance at *p* level of 0.05, and ** indicates significance at *p* level of 0.01.

**Table 4:** ANOVA on the GLM model for leaf area index (LAI), main vine length and thick stem as a function of irrigation strategies (W), planting pattern (M), and irrigation strategies × planting pattern (W × M).

Test of Significance (*F* Value)									
	LAI			Main Vine Length			Thick Stem		
	2020	2021	2022	2020	2021	2022	2020	2021	2022
Irrigation (W)	1.484	8.495 *	10.498 *	0.445	6.498 *	21.092 *	13.453 *	17.352 *	10.450 *
Planting pattern (M)	0.873	12.594 *	6.794 *	1.000	13.204 *	39.481 **	1.790	9.464 *	11.245 *
W × M	1.349	0.584	2.541 *	0.784	1.552 *	2.466 *	0.583	2.654 *	1.375 *

* indicates significance at *p* level of 0.05, and ** indicates significance at *p* level of 0.01.

**Table 5:** Yield, total soluble solids, and average weight affected by planting patterns (M) and irrigation strategies (W) and their interaction (W × M) in 2020, 2021, 2022. Different letters indicated significant differences among different treatments (*p* < 0.05) by LSD test.

	Yield (kg ha^−1^)			Total Soluble Solids (%)			Average Weight (kg)		
Treatment	2020	2021	2022	2020	2021	2022	2020	2021	2022
AFW1	35,743.5 ± 400.94 ab	34,424 ± 369.21 bc	33,280.83 ± 208.12 ab	13.2 ± 0.33 a	12.47 ± 0.62 ab	13.46 ± 0.44 a	6.79 ± 0.49 b	6.68 ± 0.56 b	5.81 ± 0.41 c
AFW2	37,126.67 ± 336.19 a	35,955.83 ± 579.21 ab	34,294.33 ± 204.63 ab	12.45 ± 0.44 bc	10.87 ± 0.62 bc	11.42 ± 0.11 c	8.29 ± 0.84 ab	6.85 ± 1.01 b	7.03 ± 0.63 c
AFW3	36,314.83 ± 441.42 ab	37,354.67 ± 264.05 a	34,858.83 ± 634.19 ab	11.4 ± 0.08 d	11.37 ± 0.53 c	11.6 ± 0.31 c	7.65 ± 0.89 a	7.42 ± 0.24 b	7.63 ± 0.41 b
SCW1	34,238.67 ± 146.06 b	33,986.67 ± 265.8 c	32,608 ± 292.86 b	12.77 ± 0.29 ab	12.83 ± 0.61 a	12.67 ± 0.12 b	7.27 ± 1 a	6.88 ± 0.97 a	5.97 ± 0.52 a
SCW2	37,288 ± 396.65 a	37,354.83 ± 544.56 a	36,484.83 ± 456.93 a	12.43 ± 0.17 bc	11.93 ± 0.45 abc	11.2 ± 0.08 c	8.45 ± 0.54 ab	8.15 ± 0.73 ab	7.71 ± 0.52 a
SCW3	36,684.17 ± 525.19 ab	35,627.5 ± 716.25 b	35,957.5 ± 677.68 a	11.87 ± 0.17 cd	11.47 ± 0.49 bc	10.43 ± 0.17 d	7.73 ± 0.49 ab	7.11 ± 0.59 ab	7.24 ± 0.4 b
**Test of significance (*F* value)**									
Irrigation (W)	36.464 **	13.433 **	17.484 **	22.412 **	2.523	15.364 **	2.584	2.593	43.535 **
Planting pattern (M)	1.594	2.879	4.362 *	0.483	3.693	9.461 **	3.211	1.240	2.132
W × M	4.542 **	2.081 **	2.330 **	3.190 **	1.599 **	2.046 **	4.324 **	0.574	1.534

* indicates significance at *p* level of 0.05, and ** indicates significance at *p* level of 0.01.

**Table 6:** Land equivalent ratio (LER) of watermelon and apple tree in three study years.

Irrigation Strategies	LER		
	2020	2021	2022
W1	-	1.07	1.21
W2	-	1.13	1.23
W3	-	1.14	1.26

## Data Availability

Data are contained within the article.
